# Microbiome diversity in *Diaphorina citri* populations from Kenya and Tanzania shows links to China

**DOI:** 10.1371/journal.pone.0235348

**Published:** 2020-06-26

**Authors:** Inusa J. Ajene, Fathiya M. Khamis, Barbara van Asch, Gerhard Pietersen, Brenda A. Rasowo, Fidelis L. Ombura, Anne W. Wairimu, Komivi S. Akutse, Mamoudou Sétamou, Samira Mohamed, Sunday Ekesi

**Affiliations:** 1 International Center of Insect Physiology and Ecology, Nairobi, Kenya; 2 Department of Genetics, Stellenbosch University, Stellenbosch, South Africa; 3 Department of Crop Protection, Ahmadu Bello University, Zaria, Nigeria; 4 Texas A&M University, Kingsville Citrus Center, Weslaco, Texas, United States of America; US Department of Agriculture, UNITED STATES

## Abstract

The Asian citrus psyllid (*Diaphorina citri*) is a key pest of *Citrus* spp. worldwide, as it acts as a vector for “*Candidatus* Liberibacter asiaticus (Las)”, the bacterial pathogen associated with the destructive Huanglongbing (HLB) disease. Recent detection of *D*. *citri* in Africa and reports of Las-associated HLB in Ethiopia suggest that the citrus industry on the continent is under imminent threat. Endosymbionts and gut bacteria play key roles in the biology of arthropods, especially with regards to vector-pathogen interactions and resistance to antibiotics. Thus, we aim to profile the bacterial genera and to identify antibiotic resistance genes within the microbiome of different populations worldwide of *D*. *citri*. The metagenome of *D*. *citri* was sequenced using the Oxford Nanopore full-length 16S metagenomics protocol, and the “What’s in my pot” (WIMP) analysis pipeline. Microbial diversity within and between *D*. *citri* populations was assessed, and antibiotic resistance genes were identified using the WIMP-ARMA workflow. The most abundant genera were key endosymbionts of *D*. *citri* (“*Candidatus* Carsonella”, “*Candidatus* Profftella”, and *Wolbachia*). The Shannon diversity index showed that *D*. *citri* from Tanzania had the highest diversity of bacterial genera (1.92), and *D*. *citri* from China had the lowest (1.34). The Bray-Curtis dissimilarity showed that China and Kenya represented the most diverged populations, while the populations from Kenya and Tanzania were the least diverged. The WIMP-ARMA analyses generated 48 CARD genes from 13 bacterial species in each of the populations. Spectinomycin resistance genes were the most frequently found, with an average of 65.98% in all the populations. These findings add to the knowledge on the diversity of the African *D*. *citri* populations and the probable introduction source of the psyllid in these African countries.

## Introduction

The Asian citrus psyllid, *Diaphorina citri* Kuwayama (Hemiptera: Psyllidae) is a major pest of citrus plants, as it transmits “*Candidatus* Liberibacter asiaticus” (Las), the pathogen associated with Huanglongbing (HLB) worldwide [1]. *Diaphorina citri* is native to Asia and widely distributed in southern Asia, but it has also been reported in South America, North America, and more recently, in Africa [2–5]. HLB was responsible for the destruction of several citrus industries in Asia and America in the past decade [6]. As HLB is currently incurable, prevention of further spread of the disease calls for the management of the psyllid vector. The most commonly employed method for the control of *D*. *citri* relies on the use of synthetic insecticides [7]. Unfortunately, insecticides are not species-specific and have negative impact on the environment. Therefore, synthetic chemicals should be complemented or replaced by alternative methods, such as biological control using parasitoids, and the manipulation of endosymbionts. The presence of endosymbiotic microbes within the insect has been shown to benefit the host, and their loss leads to decreased fitness [8,9]. Endosymbionts play a critical role in the reproductive fitness of insects and their ability to transmit pathogens. The presence of the key endosymbionts and their locations within the insect tissue gives insight into their probable effects on the acquisition and transmission of Las by *D*. *citri* for example, “*Candidatus* Profftella” and “*Candidatus* Carsonella” which are vertically transferred from mother to offspring, have been shown to be localized in the bacteriome, with “*Candidatus* Carsonella” localizing to the outer layer in the cytoplasm of the bacteriocytes, while “*Candidatus* Profftella” localizes to the inner syncytial cytoplasm while *Wolbachia pipientis* wDi is localized the outside layer of the bacteriome [10]. Furthermore, *Wolbachia pipientis* is present in the Malpighian tubules and within midgut epithelial cells, the outer membrane cells of the scrotal capsule, testes, and accessory glands [10]. Bacterial symbionts have been considered as a potential tool to manipulate reproduction of *D*. *citri* or suppress transmission of Las to citrus plants [11]. Studies on the endosymbiont densities in different tissues and Las in healthy and Las-exposed, male and female *D*. *citri* individuals showed variation among the bacterial densities, for example, and Las is known to have different effects on male and female *D*. *citri* endosymbionts [10]. Key endosymbionts of *D*. *citri* such as *Wolbachia pipientis wDi*, “*Candidatus* Profftella” and “*Candidatus* Carsonella” have diverse roles within the insect. “*Candidatus* Carsonella” is a putative nutrition provider, while “*Candidatus* Profftella” has been reported as a defensive symbiont [12,13]. *Wolbachia* is maternally transmitted in the egg cytoplasm and performs reproductive manipulations to increase the fitness of *Wolbachia*-infected matrilines resulting in cytoplasmic incompatibility between uninfected females and infected males [13]. This activity of *Wolbachia* has been exploited as a potential control measure to decrease insect populations such as *Ceratitis capitata*, *Aedes aegypti* and *Aedes albopictus* [14–18]. The study of microbial communities through metagenomics helps to understand their interactions with arthropods, disease transmission, immunity, host resistance and insect host vector competence [19,20]. The taxonomic composition of metagenomes can be profiled using short-read high-throughput sequencing followed by mapping the reads to reference genomes, and analysis of k-mer distribution [21]. However, the taxonomic resolution of the target community to the species level is limited by read length.

Recent reports of the presence of *D*. *citri* in Tanzania and Kenya [4,5] and *“Ca*. *L*. *asiaticus”* in Ethiopia [22,23] highlighted the need to profile the metagenome diversity in the insect vector populations. The identification of key endosymbionts and other bacteria, as well as antibiotic resistance genes in the microbial community within the insect can inform the diversity between the newly introduced African populations and older populations. The main objectives of this study were to evaluate microbiome and endosymbiont diversity in *D*. *citri* populations, and to detect antibiotic resistance genes in the microbiome of the insects.

## Materials and methods

### Sample collection and DNA extraction

Collection of *D*. *citri* specimens were carried out in orchards and backyard gardens in Kenya and Tanzania from March 2017 to December 2018 (S1 Table). Adult psyllids were aspirated from citrus plants at the collection sites and stored in 96% ethanol. Adult psyllids were also obtained from citrus trees in Fuzhou, Fujian Province, (China) (S1 Table). Five female insect specimens per country collected from sweet orange (*Citrus sinensis*) trees were randomly selected for further analysis. Specimens were washed in 3% sodium hypochlorite for three seconds, rinsed thrice with distilled water to remove microbial contaminants, and stored at -80°C until DNA extraction. Total DNA was extracted from individual specimens using the Isolate II Genomic DNA kit (Bioline, London, United Kingdom), following the manufacturer’s instructions. DNA extracts were checked for quality and concentration using a Nanodrop 2000/2000c Spectrophotometer (Thermo Fischer Scientific, Wilmington, USA). DNA extracts within the A_260 nm_/A_280nm_ ratio range of 1.8 to 2.0 were eluted to a final volume of 50 μl, and used for downstream analyses.

### PCR and Sanger sequencing for identification of endosymbiont

A preliminary screening of the DNA extracts was performed using universal primers for bacterial 16S rRNA (S2 Table) [24]. The same gene region was amplified using *Wolbachia-*specific primers [25]. The sequences generated in this study were deposited in GenBank (accession numbers MN928700—MN928707). The phylogeny of the key endosymbionts of *D*. *citri* was assessed using a compilation of selected publicly available 16S sequences on GenBank. Multiple sequence alignments were performed using the MAFFT. The final sequence alignment (n = 1400 bp) was used to construct a Maximum likelihood (ML) tree to display the relationships among the microbial sequences from different regions. The ML tree was constructed using MEGA X based on p-distances under the Tamura-Nei model, with 1,000 bootstrap replicates.

### MinION sequencing

The MinION 1D^2^ sequencing chemistry (Oxford Nanopore Technologies, Inc., Oxford, UK) provides high information content in long reads (10,000–100,000 bp), resulting in longer alignments and, therefore, higher taxonomic resolution and specificity [21]. For this survey, total DNA from five individuals per country was pooled, and analysed as a single sample. Sequencing of bacterial 16S rRNA was performed on an Oxford Nanopore Technologies (ONT) MinION device using R9.4 flow cells to obtain the full-length 16S rRNA metagenome (~1,500 bp). Libraries were prepared using the SQK-RAB204 16S Barcoding kit following the ONT “16S Barcoding Kit (SQK-RAB204)” protocol, according to the manufacturer’s instructions. The library preparation PCR step was carried out in a total reaction volume of 50 μl containing 5X My Taq reaction buffer (5 mM dNTPs, 15 mM MgCl_2_, stabilizer and enhancer) (Bioline), 0.5 pmol μl^-1^ of each 16S barcode, 0.0625 U μl^-1^ MyTaq DNA polymerase (Bioline), and 10 ng μl^-1^ of DNA template. Reactions were run in a Mastercycler Nexus gradient thermal cycler (Eppendorf, Germany), under the following conditions: initial denaturation for 2 min at 95°C, followed by 35 cycles of denaturation for 30 s at 95°C, annealing for 40 s at 55°C and extension for 1 min at 72°C, and a final extension step of 10 min at 72°C. Libraries were purified, pooled and diluted to 4 nM prior to MinION sequencer runs, which ranged from 4 to 8 h.

### Base calling and read preparation

Live base calling of the MinION reads was performed using the ONT Albacore in the MinKNOW software (v19.05.0). All raw data were deposited in the NCBI database as BioProject: PRJNA598520. Quality assessment of each MinION dataset was performed during the runs via MinKNOW. The raw MinION reads collected during the sequencing runs by MinKNOW were immediately uploaded to the ONT cloud for analyses via EPI2ME (v2.59.1896509), after which base-called data were returned to the host computer, also in the form of FASTQ files. The “What’s in my Pot” (WIMP) [26] workflow classifies and identifies species in real-time. FASTQ-WIMP (v3.2.1) (24) (https://epi2me.nanoporetech.com/) was used to extract and characterize the numbers of reads and assign taxonomy. FASTQ-WIMP, which has been shown to work for long-read data [27], was used to analyze the datasets and assign taxonomic identification by comparing the reads against a NCBI database for bacteria. WIMP makes use of Centrifuge software [28], which is capable of accurately identifying reads when using databases containing multiple highly similar reference genomes, such as different strains of bacterial species. Centrifuge identifies unique segments of those genomes and builds an FM-index that can be used for efficient searches of sequenced reads.

### Bacterial diversity statistics

Direct quantitative comparison of abundances was done at the genus level using a stacked bar plot to view the cumulative read counts from the samples for each country. A minimum abundance cut-off of 0.1% was used to select the most abundant taxa in each sample. Taxa with cumulative read counts below the 0.1% cut-off were collapsed into the “Others” category. Viewing composition at higher-levels (e.g. genus) provides a better picture than at lower-levels (e.g. species), when the number of bacterial species in a community is large and diversified. Merging minor taxa helps to better visualize significant taxonomic patterns in the data. Alpha diversity statistics, which included evenness, richness and the Shannon-Wiener index, were used to determine the bacterial diversity in each sample. Beta diversity was computed using the Bray Curtis dissimilarity index [29,30] to determine the diversity of bacterial genera among the samples. Inter-population distances were visualized using principal coordinate analyses (PCoA) computed in R software v3.5.3, using the ‘*vegan’* package [31].

### Antibiotic resistance genes

MinION reads from the WIMP workflow were searched for antibiotic resistance genes (ARGs) using the ARMA workflow in EPI2ME (https://epi2me.nanoporetech.com/). The workflow is integrated with the Comprehensive Antibiotic Resistance Database (CARD) to identify ARGs. The CARD model used in this analysis was the rRNA mutation model. Depth of coverage, alignment details, and resistance profile from the CARD database were generated for specific genes.

## Results

### Identification of endosymbionts using Sanger sequencing

**Fig 1:**
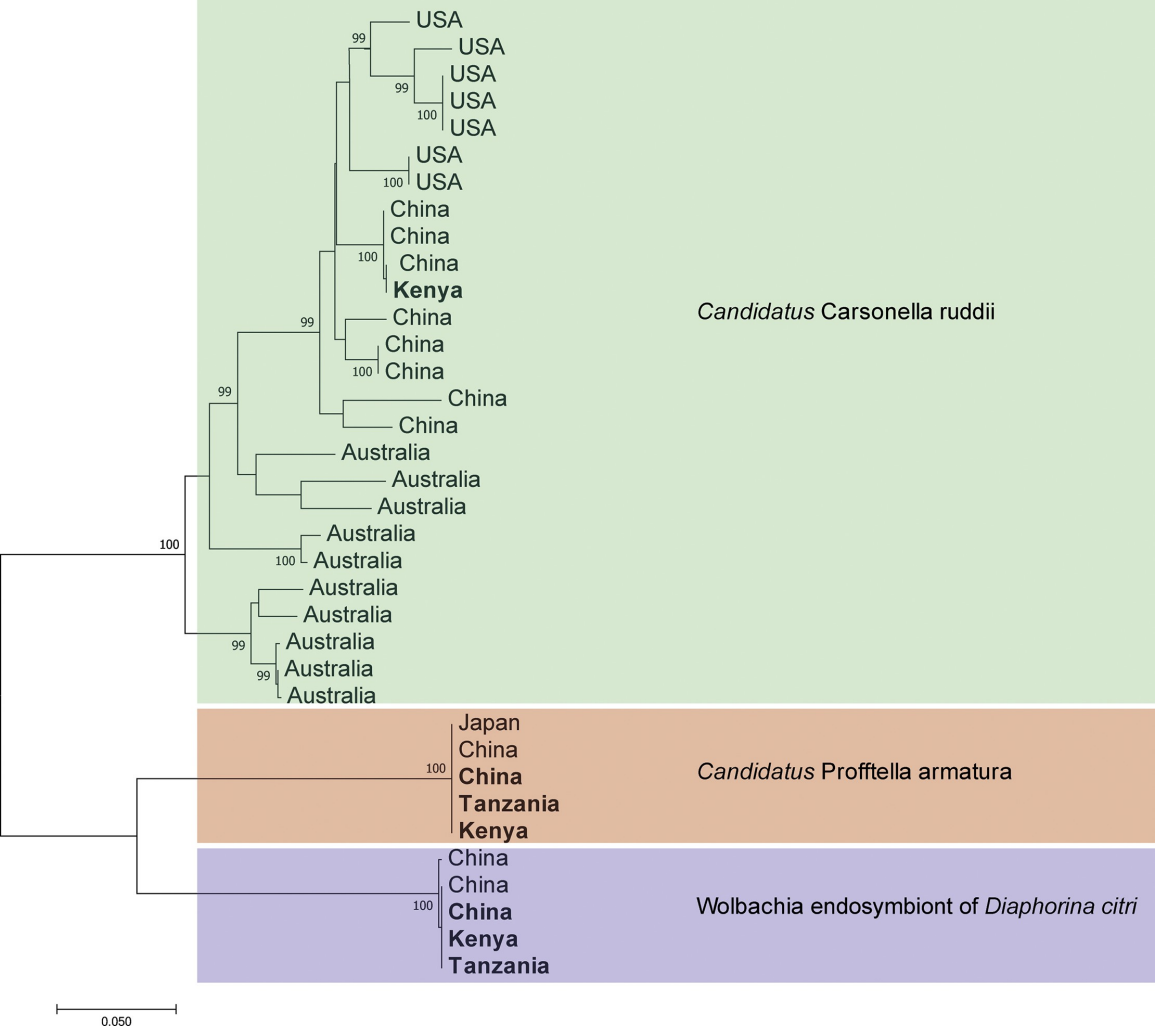
Maximum-likelihood tree based on 16S ribosomal RNA gene sequences of “*Candidatus* Carsonella ruddii”, “*Candidatus* Profftella armatura” and *Wolbachia* endosymbiont of *Diaphorina citri* from China, Kenya and Tanzania and representative sequences from publicly available sequences on GenBank. Branch support was based on 1000 bootstrap replicates. Samples from this study are in bold font.

PCR and Sanger sequencing of individual *D*. *citri* specimens using universal primers for bacteria (27F/149R) generated sequences (accession nos. MN928700—MN928703) which showed high percentage sequence similarity with *Wolbachia sp*. (100%), “*Candidatus* Profftella armatura” (100%), *Pseudomonas sp*. (97%), “*Candidatus* Carsonella ruddii” (98%) and *Enterobacteriaceae sp*. (96%), respectively (S3 Table) in all samples. The *Wolbachia*-specific primers (wspecF/wspecR) generated sequences identified as *Wolbachia sp*., based on sequence homology using BLAST. The sequences from this study (accession nos. MN928704—MN928707) had 100% identity with *Wolbachia* endosymbiont of *D*. *citri* sequences in GenBank. The Maximum-Likelihood tree was built using 16S rRNA sequences obtained from the samples in this study combined with 16S rRNA sequences of the representative sequences available in GenBank to assess the phylogeographic structure of the key endosymbionts of *D*. *citri*. The tree topology indicated that “*Candidatus* Carsonella ruddii” from this study clustered closely with “*Candidatus* Carsonella ruddii” from China while a wider variation was seen between the sequences from this study and the sequences from Australia and the USA. The “*Candidatus* Profftella” sequences from this study was closely related to the publicly available sequences obtained from China and Japan. The same trend was observed with the *Wolbachia* sequences obtained from this study (Fig 1).

### Full length 16S metagenome profiling

#### Library size and cumulative abundance

**Fig 2:**
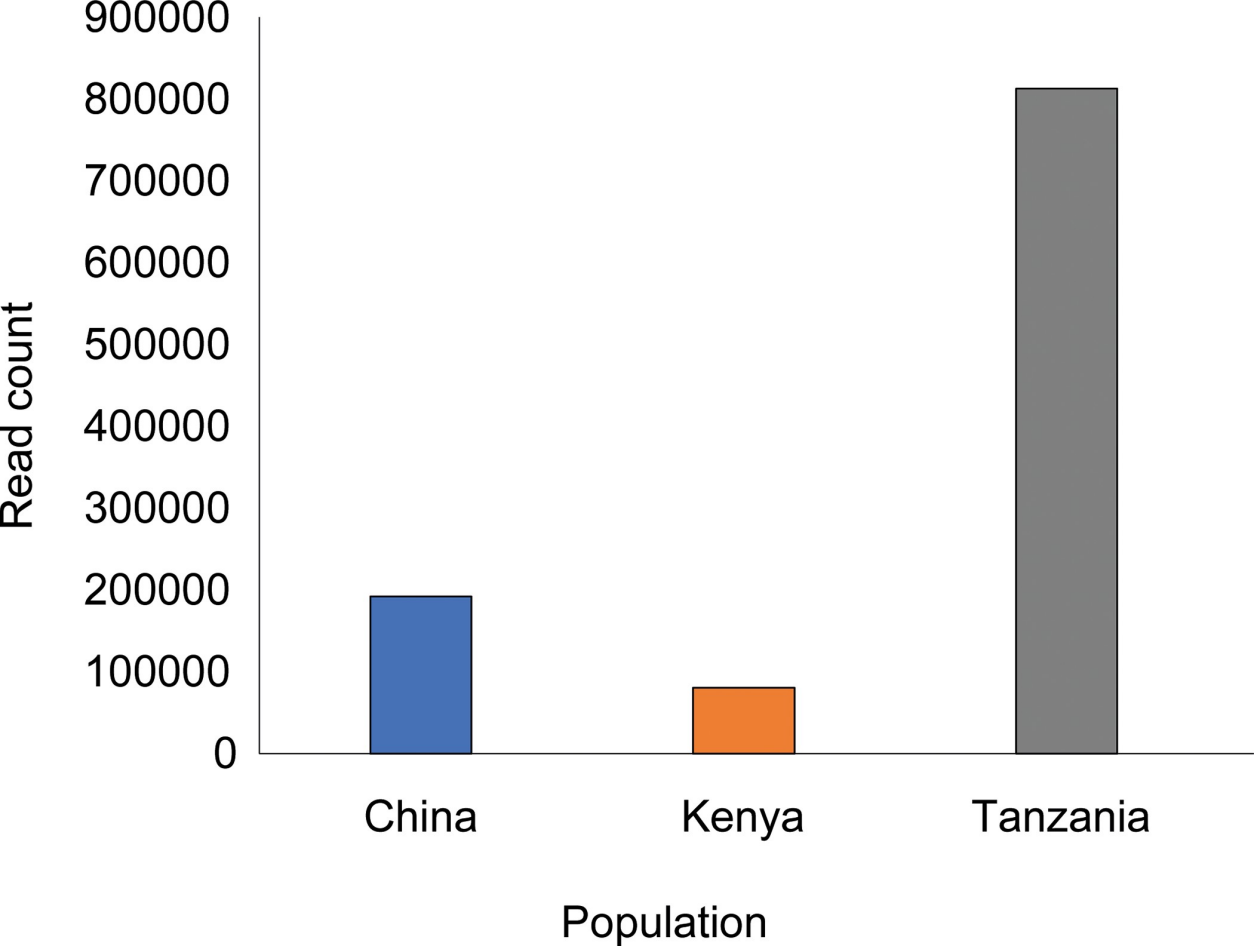
Overview of 16S rRNA library size for MinION sequencing of five pooled *Diaphorina citri* individuals collected from each world region (China, Kenya and Tanzania).

**Fig 3:**
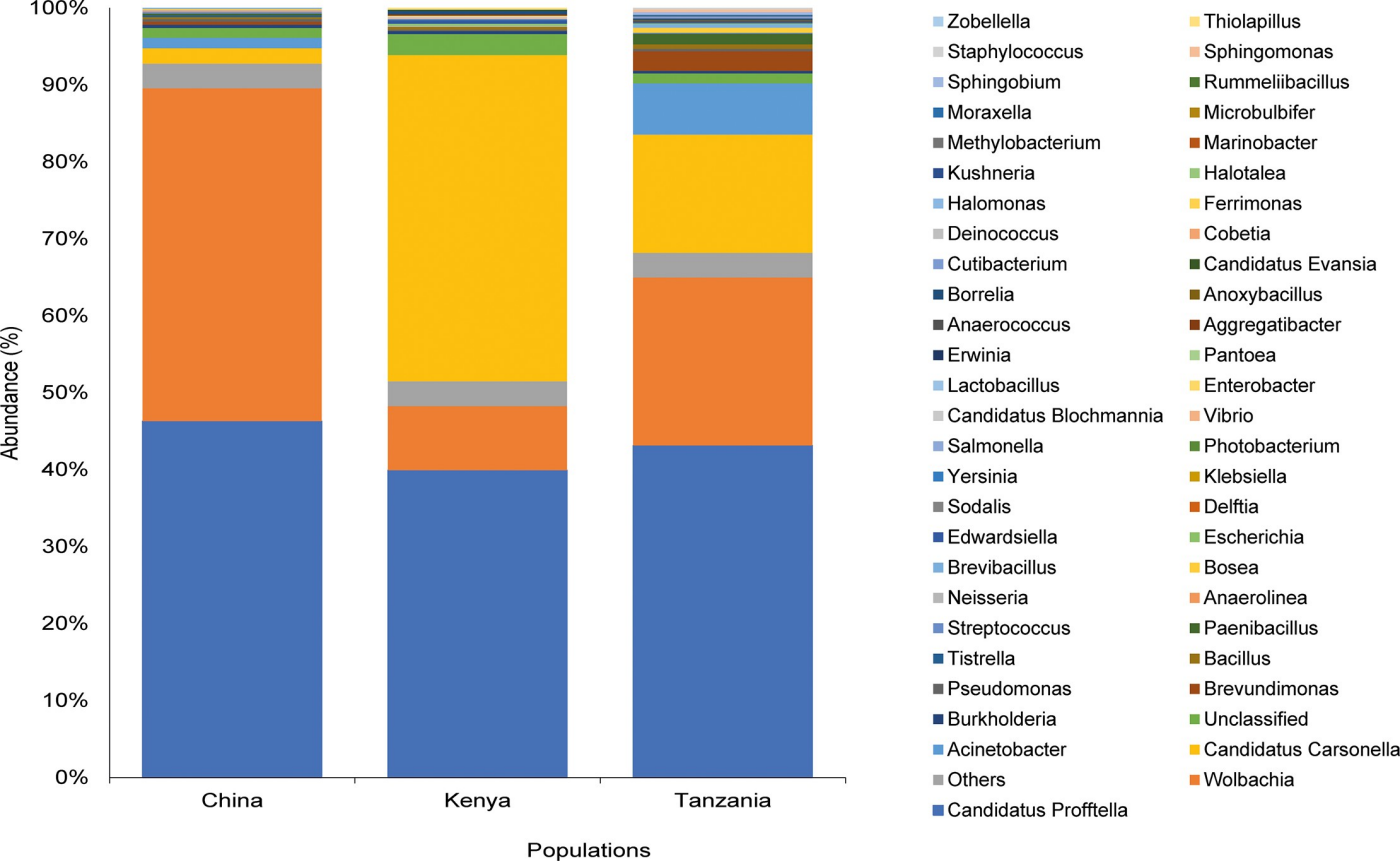
Taxonomic composition and percentage of reads of bacteria at the genus level of the bacterial community in *Diaphorina citri* from China, Kenya and Tanzania, using Stacked Bar plot. Taxa with cumulative read counts below the cut-off value of 0.1% were collapsed into ‘Others’ category.

Library size was the largest for *D*. *citri* from Tanzania (812,730 reads), followed by those from China (192,136 reads) and Kenya (80,521 reads) (Fig 2). The taxonomic composition of the samples and the cumulative abundance of the bacterial genomes present in *D*. *citri* from the different world regions are presented in Fig 3 and showed that the most abundant genera in China were “*Candidatus* Profftella” making up 46.32% of the bacterial population, *Wolbachia* (43.24%) and “*Candidatus* Carsonella” (1.99%). In Kenya, the most abundant genera were “*Candidatus* Carsonella” (42.41%), “*Candidatus* Profftella” (39.94%) and *Wolbachia* (8.33%). In Tanzania, the most abundant genera were “*Candidatus* Profftella” (43.15%), *Wolbachia* (21.83%) and “*Candidatus* Carsonella” (15.36%) (Fig 3).

### Bacterial community profiling

#### Alpha diversity

**Fig 4:**
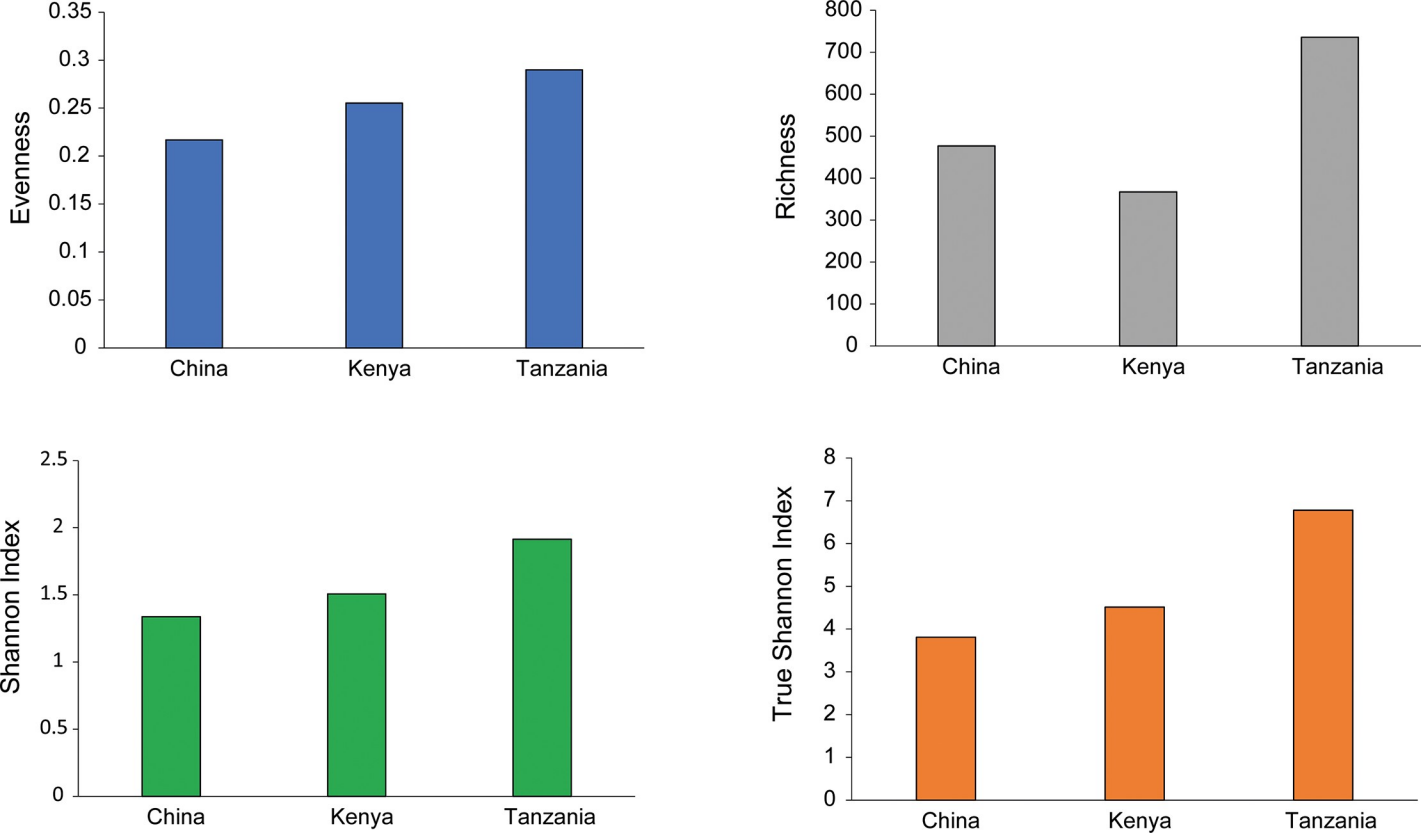
Alpha-diversity measures using evenness, Shannon diversity index, and species richness at the genus level across *Diaphorina citri* collected in different world regions (China, Kenya and Tanzania). The samples were composed by five pooled individual insects, and are represented on the X-axis, and their estimated diversity is represented on the Y-axis.

The analysis showed that *D*. *citri* from Tanzania had the highest species richness (736), followed by China (477) and Kenya (367) (S4 Table). The Shannon diversity index showed that *D*. *citri* from Tanzania had the highest diversity of bacterial genera (1.92), followed by Kenya (1.51) and China (1.34) (Fig 4). The effective diversity (True-Shannon) showed that *D*. *citri* from Tanzania had the highest effective number of species (6.78), followed by Kenya (4.51), and China (3.81) (S4 Table).

#### Beta diversity

**Fig 5:**
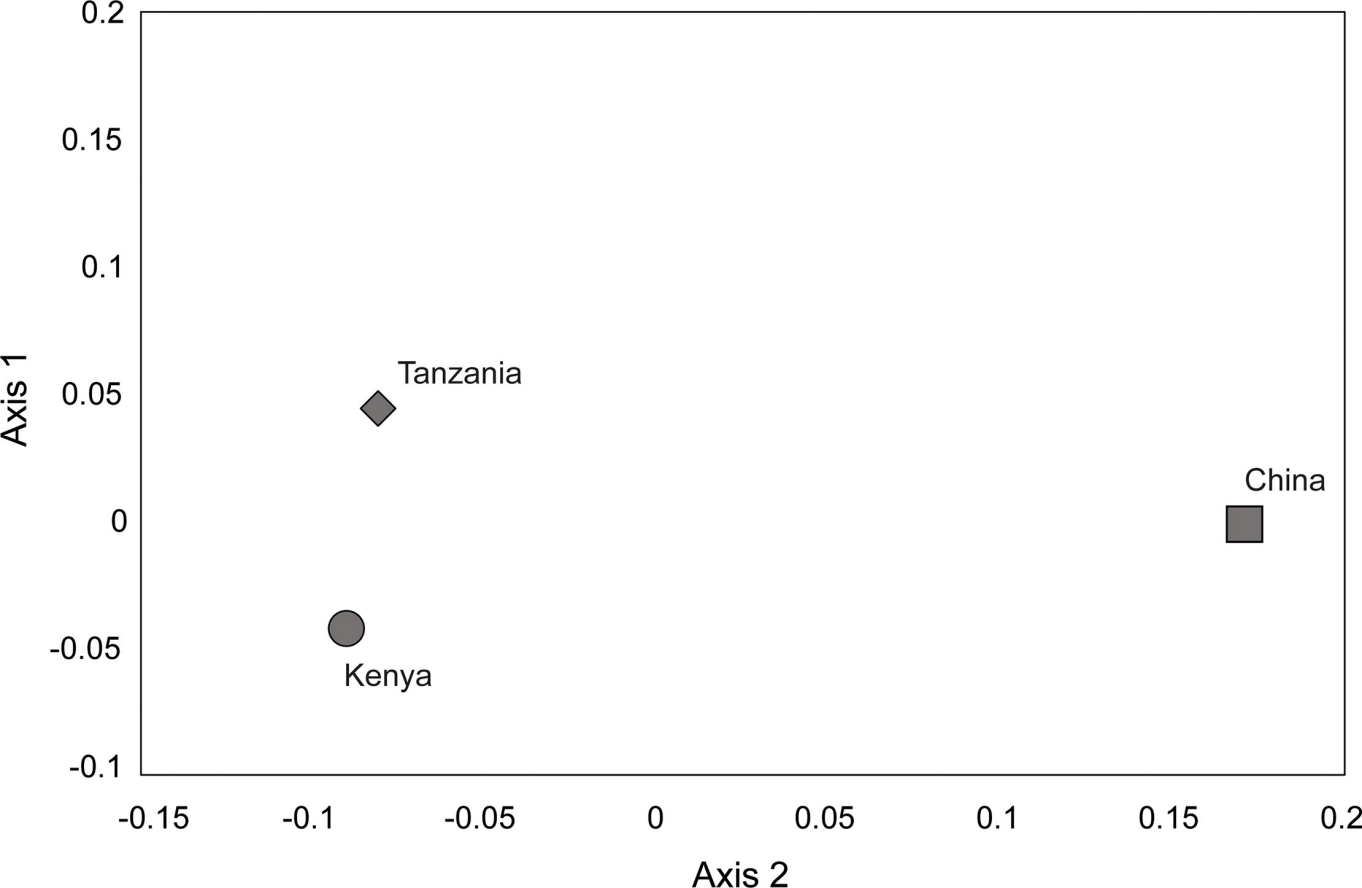
Two-dimensional principal coordinate analyses plot of the beta diversity of bacterial genera in *Diaphorina citri* collected from different countries, estimated using the Bray Curtis dissimilarity index showing.

The highest value in interpopulation diversity, as estimated with the Bray Curtis dissimilarity index, was obtained between Kenya and China (26.43%) (Table 1) while the lowest interpopulation diversity was observed between Kenya and Tanzania (8.72%), (Fig 5, Table 1).

**Table 1:** Interpopulation beta diversity (%) in the metagenomes of *Diaphorina citri* from different world regions, as estimated using the Bray Curtis dissimilarity index.

	Dissimilarity (%)		
World region	China	Kenya	Tanzania
China	-		
Kenya	26.43	-	
Tanzania	25.61	8.72	-

### Abundance of antibiotic resistance genes in *Diaphorina citri*

**Fig 6:**
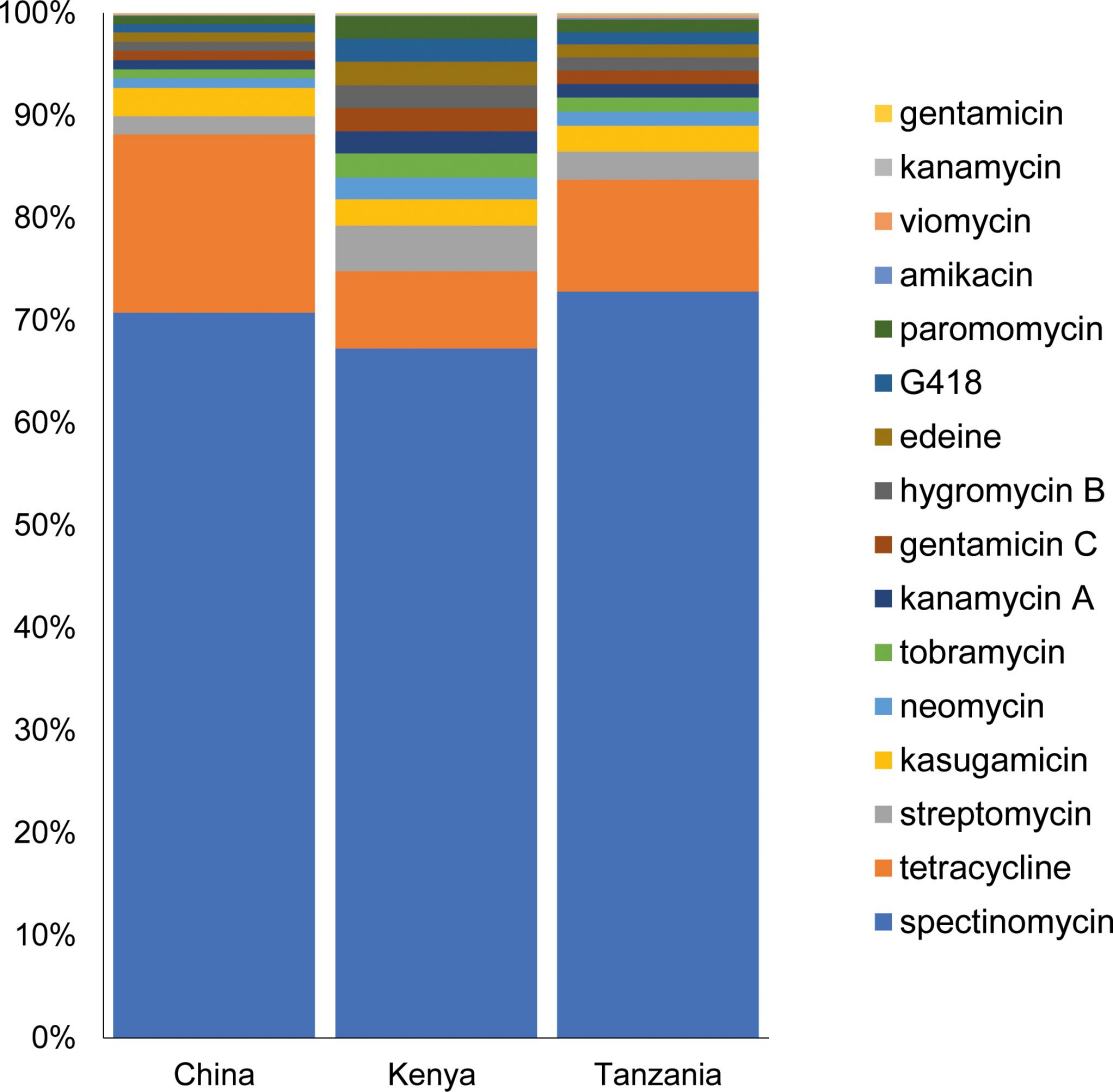
Relative abundance of genes for resistance to antibiotics identified in the microbiome of *Diaphorina citri* from China, Kenya and Tanzania.

The number of 16S rRNA reads analysed ranged from 44,455 (Kenya) to 399,992 (Tanzania), with an overall average accuracy of the read alignments to the CARD of 75.45% (S5 Table). The identified ARGs conferred resistance to 16 antibiotic compounds (Table 2). Among the detected ARGs in the microbiome of *D*. *citri*, spectinomycin resistance genes were the most abundant, making up 72.82%, 70.75% and 67.29% of the total ARGs found in Tanzania, China and Kenya respectively. This was followed by tetracycline resistance genes with 17.42%, 10.95% and 7.55% detected in China, Tanzania and Kenya respectively. Streptomycin resistance genes in Kenya (4.43%) and Tanzania (2.71%), and kasugamicin resistance genes were the third most abundant in China (2.74%) (Fig 6).

**Table 2:** Antibiotic resistance genes found in the microbiome of the citrus psyllid *Diaphorina citri*, as obtained from comprehensive antibiotic resistance database.

Taxon	Gene	Conferred antibiotic resistance
*Borrelia burgdorferi*	*Borrelia burgdorferi* 16S rRNA mutation	resistance to gentamicin, kanamycin and spectinomycin
*Chlamydia psittaci* 6BC	*Chlamydophila psittaci* 16S rRNA mutation	resistance to spectinomycin
*Escherichia coli* K-12	*Escherichia coli* 16S rRNA mutation	resistance to edeine
	*Escherichia coli* 16S rRNA mutation in the rrnB gene	resistance to spectinomycin, streptomycin and tetracycline
	*Escherichia coli* 16S rRNA mutation in the rrsB gene	resistance to G418, gentamicin C, kanamycin A, neomycin, paromomycin, spectinomycin, streptomycin, tetracycline and tobramycin
	*Escherichia coli* 16S rRNA mutation in the rrsC gene	resistance to kasugamicin
	*Escherichia coli* 16S rRNA mutation in the rrsH gene	resistance to spectinomycin
*Helicobacter pylori* 26695	*Helicobacter pylori* 16S rRNA mutation	resistance to tetracycline
*Mycobacterium abscessus*	*Mycobacterium abscessus* 16S rRNA mutation	resistance to amikacin, gentamicin, kanamycin, neomycin and tobramycin
*Mycobacterium*	*Mycobacterium chelonae* 16S rRNA mutation	resistance to amikacin, gentamicin C, kanamycin A, neomycin and tobramycin
*Mycobacterium smegmatis str*. *MC2 155*	*Mycobacterium smegmatis* 16S rRNA mutation in the rrsA gene	resistance to hygromycin B, kanamycin A, neomycin and viomycin
	*Mycobacterium smegmatis* 16S rRNA mutation in the rrsB gene	resistance to hygromycin B, kanamycin A, neomycin, streptomycin and viomycin
*Mycobacterium tuberculosis H37Rv*	*Mycobacterium tuberculosis* 16S rRNA mutation	resistance to amikacin, kanamycin, streptomycin and viomycin
*Neisseria gonorrhoeae*	*Neisseria gonorrhoeae* 16S rRNA mutation	resistance to spectinomycin
*Neisseria meningitidis*	*Neisseria meningitidis* 16S rRNA mutation	resistance to spectinomycin
*Pasteurella multocida 36950*	*Pasteurella multocida* 16S rRNA mutation	resistance to spectinomycin
*Propionibacterium acnes*	*Propionibacterium acnes* 16S rRNA mutation	resistance to tetracycline
*Salmonella enterica* subsp. *salamae*	*Salmonella enterica* serovar *Typhimurium* 16S rRNA mutation in the rrsD gene	resistance to spectinomycin

## Discussion

The standard methodology in metagenomics involves sequencing and annotation of whole genomes extracted directly from samples to profile the microbial composition [32]. Efforts to use this technology to study diverse environmental communities have been limited [33], and there has been a limited assessment of the best strategies for data analysis in nanopore-based environmental metagenomics [21]. In this study, we assessed the microbiome diversity and presence of bacterial antibiotic resistance genes in a pool of five *D*. *citri* specimens collected from each of three countries (China, Kenya and Tanzania), using ONT long-read sequencing of the full length 16S metagenome. The nanopore sequencing resulted in good read coverage (up to 800,000 reads). The bacterial community profiling in this study relied on the cumulative read count generated from the FASTQ-WIMP and is presented as a percentage of reads of specific bacterial genera amongst reads to all bacterial genera.

The preliminary screening of bacteria in *D*. *citri* using Sanger sequencing with universal and *Wolbachia*-specific primers revealed the presence of the key endosymbionts “*Candidatus* Carsonella”, “*Candidatus* Profftella” and *Wolbachia*, along with other bacterial species. The full-length 16S metagenome analysis correlated with the preliminary screening, showing that the dominant genera in the three samples representing each country were similarly comprised of “*Candidatus* Profftella”, “*Candidatus* Carsonella” and *Wolbachia*, which are key endosymbionts of *D*. *citri* [12]. This agrees with previous studies on the microbiota of *D*. *citri* which identified “*Candidatus* Profftella”, “*Candidatus* Carsonella” and *Wolbachia* as the endosymbionts with the highest composition [10,34–36].

*“Candidatus* Profftella” had the highest abundance in the three populations tested, while *Wolbachia* had the second-highest abundance in Tanzania and third highest abundance in Kenya. Previously, “*Candidatus* Profftella” was reported to be present in all *D*. *citri* populations [12]. The difference in the microbial population composition between the Kenyan and Tanzanian population could be a result of the age of the psyllids as previous reports have shown that gender and age could be factors affecting the densities of endosymbionts between populations [36]. Our study specifically considered the microbiota composition in females partly because it has been reported that “*Candidatus* Profftella” and “*Candidatus* Carsonella” show an increase in the female ovaries in the presence of Las and thus a potentially greater effect on the reproductive ability of *D*. *citri*. Also, higher densities of “*Candidatus* Profftella” and “*Candidatus* Carsonella” in the bacteriome of Las-infected females as compared to Las-infected males have been reported [10]. This could account for the higher density of the two endosymbionts seen in the current study.

*Wolbachia* is known to cause cytoplasmic incompatibility between uninfected females and infected males resulting in increased infected lineages in the host population [13]. The ability of *Wolbachia* to cause incompatibility in insects can be exploited as a potential strategy to control *D*. *citri* in addition to other control methods, as has been shown in the Mediterranean fruit fly and the olive fruit fly [37,38]. Additionally, *Wolbachia* can play a crucial role in the reduction in transmission efficiency of “*Ca*. L. asiaticus” by *D*. *citri* by causing a reduction in the production of saliva in the psyllid, as the Liberibacter is transmitted through the saliva during feeding. This has been shown to be effective in mosquitoes where *Wolbachia*-infected *Aedes aegypti* produce smaller volumes of saliva [39]. Furthermore, as *Wolbachia* has been shown to display an increased density in Las-exposed males, the possibility of vector manipulation using Wolbachia that may affect the ability of *D*. *citri* to transmit Las has been hypothesized [10].

Our study showed that *D*. *citri* from Tanzania had the highest degree of homogeneity (high evenness index), while the population from China was the most heterogeneous. This suggests that all bacterial species within the *D*. *citri* from Tanzania have a higher rate of equal abundance within the microbiome compared to the other populations, as evenness is a measure of the homogeneity in terms of species abundance [40]. Species richness (a measure of the number of species or other taxonomic levels present in a population) showed that the *D*. *citri* from Tanzania had the most bacterial taxa and is therefore likely to harbor a more ecologically complex microbiome. Criticisms of the Shannon–Wiener index due to the log- transformation of species proportion values, which weakens the discriminating power of the index [41], informed the use of True-Shannon index (effective number of species) [42] to yield an effective number of species in this study. The True-Shannon diversity or the effective number of species, which considers species richness, as well as the dominance of a species, is a more complex measure of how many different types of taxa are present in a population [42]. The True-Shannon diversity showed that *D*. *citri* from Tanzania had the most diverse microbiome, followed by Kenya. *Diaphorina citri* from China, which was richer in bacterial species than *D*. *citri* from Kenya, had lower diversity. The negative correlation between Shannon diversity and richness observed in this study agrees with the report of the negative correlation between species richness and Shannon-Weiner diversity [40]. The species richness and evenness were positively correlated in this study. Wilsey [43] also found that species richness and evenness were positively correlated within invertebrate communities.

Interpopulation comparison, as estimated by Bray-Curtis dissimilarity index, showed that *D*. *citri* microbiomes had the highest diversity between the Kenya and China. Additionally, the diversity between the *D*. *citri* metagenome from China and Tanzania was similar to the diversity between China and Kenya. Overall, the alpha and beta diversity of the metagenome of *D*. *citri* support the hypothesis of a close genetic relationship between the African and the Chinese *D*. *citri*. Furthermore, the phylogeny of the key endosymbionts showed close relationship between the *D*. *citri* from Kenya/Tanzania and *D*. *citri* from China in relation to *D*. *citri* from other regions. Most evident was the clustering of the “*Candidatus* Carsonella rudii” sequences where significant variation was seen between sequences from Australia/USA and the sequences from Kenya and China. Previous studies on the endosymbiont variation in *D*. *citri* have shown that the distribution of “*Candidatus* Carsonella ruddii” had a strong geographical structure in China [35], thereby informing the inference of a possible invasion route of *D*. *citri* from China into Eastern Africa. This highlights the need for further studies on the resolution of the origins of *D*. *citri* in Africa by assessing the population structure and genetic diversity using bi-parentally transmitted microsatellite markers.

The WIMP-ARMA workflow generated 48 CARD genes from 13 bacterial species in each of the samples. The identified genes for antibiotic resistance conferred resistance to 16 antibiotic compounds belonging to different groups: aminoglycosides (amikacin, gentamicin, gentamicin C, hygromycin B, kanamycin, kanamycin A, kasugamicin, neomycin, paromomycin, spectinomycin, streptomycin and tobramycin), pentapeptides (edeine), tetracycline, and tuberactinomycin (viomycin), in varying frequency across the three countries. The most abundant type of antibiotic resistance gene was spectinomycin resistance, across all the samples from each country, followed by tetracycline resistance. Antibiotics have been used in several countries in attempts to control HLB [44], with positive results of reduced symptoms in the treated trees as evaluated in China, Reunion Island, and the Philippines [45,46], and also on “*Candidatus* Liberibacter africanus” infected trees in South Africa [47]. However, the use of antibiotics may have accelerated the development of resistance genes within the gut microbiome in *D*. *citri* as a response to selective pressure [48]. For example, tetracycline resistance genes were present in all samples tested in our study, and tetracycline has been used in direct injection into the trunks of HLB-affected citrus trees in China and India [45,46]. The effect of antibiotics on the microbiome of insects is a cause for concern, since the microbiome may serve as reservoirs for resistance genes that can be transferred to pathogens [49–51], as well as to the host plant [52]. Furthermore, the use of antibiotics in controlling infections by pathogens also impacts beneficial bacteria since the selective pressure caused by an antibiotic can cause the buildup of resistance determinants within the microbiome community members [52].

The combination of the variation shown in the phylogenetic reconstruction of the 16S sequences, the microbiome composition and diversity as well as the composition of antimicrobial resistance genes identified in this study presents a case for the probable route of invasion of the *D*. *citri* into Kenya and Tanzania from China. Although, further research into the genetic diversity of these populations is required to confirm the origins. Furthermore, the elucidation of the endosymbionts, and microbiome community and its ARGs in *D*. *citri* provide valuable information on potential integrated pest control measures which can be used to curb the spread of the psyllid and its ability to transmit diseases. For example, manipulation of cytoplasmic incompatibility conferred by *Wolbachia* can be used to interrupt the capacity of the insect to transmit diseases [53–56], in addition to strategies aimed at reducing “*Candidatus* Profftella" within the psyllid in order to enhance predation. “*Candidatus* Profftella” is responsible for the synthesis of diaphorin (a bioactive polyketide), which creates inhibitory effects on predators [12,13]. There is potential that control measures that decrease “*Candidatus* Profftella” within *D*. *citri* might increase the susceptibility of the insect to predators. Furthermore, the influence of *Wolbachia* on the increased susceptibility of the psyllid to parasitoids [57] can be exploited.

These factors, combined with knowledge of the possible transfer of ARGs from the psyllid to the plant, can inform integrated pest control strategies. Therefore, a comprehensive assessment of the role of “*Candidatus* Profftella” and *Wolbachia* in *D*. *citri* should be evaluated in order to increase the potential of biological control applications using predators and *Wolbachia* induced cytoplasmic incompatibility, whilst encouraging a reduction in the use of antibiotics for the control of HLB. Furthermore, research on the population structure and genetic diversity of the citrus psyllids in Africa needs to be carried out for complementing the knowledge on the microbiome and endosymbiont composition of *D*. *citri* on the continent. These findings are critical to the implementation of integrated pest management strategies to curb the rapid spread of the pest and the associated pathogen *“Candidatus* Liberibacter asiaticus” to other parts of Africa.

## Supporting information

S1 Table Sampling locations of the citrus psyllid *Diaphorina citri* in China, Kenya and Tanzania. (DOCX)

S2 Table Universal 16S primers and *Wolbachia*-specific (in bold) primers used in the PCR amplification and bidirectional sequencing of endosymbionts of the citrus psyllid *Diaphorina citri*. (DOCX)

S3 Table List of publicly available sequences with homology to the 16S sequences of *Diaphorina citri* from this study using 27F/148R primers. (DOCX)

S4 Table Alpha diversity statistics for the bacterial metagenomes of the citrus psyllid *Diaphorina citri* collected in three countries (China, Kenya and Tanzania). (DOCX)

S5 Table Summary statistics for the analyses of the presence of genes for antibiotic resistance in the microbiome of the citrus psyllid *Diaphorina citri* in three countries, obtained from the ARMA workflow in EPI2ME. (DOCX)
