# Supplementary material for: Microbiome diversity in Diaphorina citri populations from Kenya and Tanzania shows links to China
Source: PLoS One. 2020 Jun 26;15(6):e0235348. doi: 10.1371/journal.pone.0235348 (PMC7319306; doi:10.1371/journal.pone.0235348)
Supplement: S1 Table — (DOCX) [file pone.0235348.s001.docx]

**S1 Table. Sampling locations of the citrus psyllid *Diaphorina citri* in China, Kenya and Tanzania*.***

| Country | Location | Latitude | Longitude | Citrus system |
| --- | --- | --- | --- | --- |
| China | Fuzhou | 26.07877 | 119.2969 | Research orchard |
| Tanzania | Mafiga | -5.22041 | 37.6593 | Small scale orchard |
|  | Mikese | -4.93511 | 39.1258 | Backyard garden |
|  | Mlali | -5.15616 | 37.6626 | Small scale orchard |
| Kenya | Awasi | 0.16696 | 35.0844 | Small scale rural farm |
|  | Koitamburot | -0.20730 | 35.1926 | Small scale rural farm |
|  | Lungalunga | -4.56247 | 39.1221 | Small scale rural farm |
|  | Soin | 0.09243 | 35.1469 | Small scale rural farm |
