# Supplementary material for: Microbiome diversity in Diaphorina citri populations from Kenya and Tanzania shows links to China
Source: PLoS One. 2020 Jun 26;15(6):e0235348. doi: 10.1371/journal.pone.0235348 (PMC7319306; doi:10.1371/journal.pone.0235348)
Supplement: S2 Table — (DOCX) [file pone.0235348.s002.docx]

**S2 Table. Universal 16S primers and *Wolbachia*-specific (in bold) primers used in the PCR amplification and bidirectional sequencing of endosymbionts of the citrus psyllid *Diaphorina citri.***

| Primer | Primer sequence (5’-3’) | Reference |
| --- | --- | --- |
| 27F | AGAGTTTGATCCTGGCTCAG | Hocquellet et al. 1999 |
| 148R | TACGGTACCTTGTTACGACTT | Hocquellet et al. 1999 |
| **wspecF** | **CATACCTATTCGAAGGGATAG** | Werren and Windsor, 2000 |
| **wspecR** | **AGCTTCGAGTGAAACCAATTC** | Werren and Windsor, 2000 |
