# Supplementary material for: Microbiome diversity in Diaphorina citri populations from Kenya and Tanzania shows links to China
Source: PLoS One. 2020 Jun 26;15(6):e0235348. doi: 10.1371/journal.pone.0235348 (PMC7319306; doi:10.1371/journal.pone.0235348)
Supplement: S3 Table — (DOCX) [file pone.0235348.s003.docx]

**S3 Table.** **List of publicly available sequences with homology to the 16S sequences of *Diaphorina citri* from this study using 27F/148R primers**

| Population | Taxon | GenBank accession | Percentage identity |
| --- | --- | --- | --- |
| China | *Pseudomonas sp.*  *Candidatus* Profftella armatura  *Wolbachia sp.* | KM253123  CP012591  MK277439.1 | 97%  100%  100% |
| Kenya | *Enterobacteriaceae sp*  *Candidatus* Carsonella ruddii  *Wolbachia sp* | EF088376  CP012411  MK277439.1 | 96%  98%  100% |
| Tanzania | *Candidatus* Profftella armatura  *Wolbachia sp* | EF433792  MK277439.1 | 100%  100% |
