# Supplementary material for: Microbiome diversity in Diaphorina citri populations from Kenya and Tanzania shows links to China
Source: PLoS One. 2020 Jun 26;15(6):e0235348. doi: 10.1371/journal.pone.0235348 (PMC7319306; doi:10.1371/journal.pone.0235348)
Supplement: S4 Table — (DOCX) [file pone.0235348.s004.docx]

**S4 Table. Alpha diversity statistics for the bacterial metagenomes of the citrus psyllid *Diaphorina citri* collected in four countries (Kenya, Tanzania, China and the USA).**

|  | EVENNESS | RICHNESS | SHANNON | TRUE_SHANNON |
| --- | --- | --- | --- | --- |
| China | 0.21 | 477 | 1.34 | 3.81 |
| Kenya | 0.26 | 367 | 1.51 | 4.51 |
| Tanzania | 0.29 | 736 | 1.91 | 6.78 |
