# Supplementary material for: Microbiome diversity in Diaphorina citri populations from Kenya and Tanzania shows links to China
Source: PLoS One. 2020 Jun 26;15(6):e0235348. doi: 10.1371/journal.pone.0235348 (PMC7319306; doi:10.1371/journal.pone.0235348)
Supplement: S5 Table — (DOCX) [file pone.0235348.s005.docx]

**S5 Table. Summary statistics for the analyses of the presence of genes for antibiotic resistance in the microbiome of the citrus psyllid *Diaphorina citri in four countries,* obtained from the ARMA workflow in EPI2ME.**

|  | China | Kenya | Tanzania |
| --- | --- | --- | --- |
| Reads analysed | 119,988 | 44,455 | 399,992 |
| Alignments | 112,462 | 41,227 | 379,386 |
| Average accuracy (%) | 74.40 | 74.20 | 75.40 |
| CARD genes | 48 | 48 | 48 |
